# 5,7-Dihydroxy-4-Methylcoumarin as a Functional Compound for Skin Pigmentation and Human Skin Safety

**DOI:** 10.3390/ph18040463

**Published:** 2025-03-25

**Authors:** Ye-Jin Lee, Yang Xu, Chang-Gu Hyun

**Affiliations:** Jeju Inside Agency and Cosmetic Science Center, Department of Chemistry and Cosmetics, Jeju National University, Jeju 63243, Republic of Korea; yyyyejin615@gmail.com (Y.-J.L.); iamxuyang1990@gmail.com (Y.X.)

**Keywords:** B16F10, 5,7-dihydroxy-4-methylcoumarin, hypopigmentation, melanogenesis, primary skin irritation test, signaling pathway

## Abstract

**Background/Objectives:** This study aims to investigate the effects of 5,7-dihydroxy-4-methylcoumarin (5,7D-4MC) on melanogenesis in B16F10 murine melanoma cells and to evaluate its safety as a potential ingredient for functional cosmetics and therapeutic agents targeting pigmentation-related disorders. **Method:** The cytotoxicity of 5,7D-4MC was assessed using an MTT assay, and melanin content and tyrosinase activity were measured at different concentrations (25, 50, 100 µM). Western blot analyses were conducted to evaluate the expression of key melanogenesis-related proteins (TYR, TRP-1, TRP-2, and MITF) and to investigate the regulation of major signaling pathways, including PKA/cAMP, GSK3β, and PI3K/AKT. Additionally, a human primary skin irritation test was performed on 32 participants to assess the dermatological safety of 5,7D-4MC. **Results:** 5,7D-4MC did not affect cell viability at concentrations below 100 µM and significantly promoted melanin production in a dose-dependent manner. Tyrosinase activity and the expression levels of melanogenic proteins increased significantly following 5,7D-4MC treatment. PKA and GSK3β pathways were activated, while the PI3K/AKT pathway was downregulated. The skin irritation test showed that 5,7D-4MC exhibited low irritation potential at concentrations of 50 µM and 100 µM. **Conclusions:** 5,7D-4MC enhances melanogenesis and demonstrates low skin irritation, making it a promising candidate for therapeutic applications in treating hypopigmentation disorders, such as vitiligo, as well as a functional cosmetic ingredient. However, further studies involving human melanocytes and clinical trials are required to validate their efficacy.

## 1. Introduction

Skin pigmentation disorders can be broadly categorized into hyperpigmentation (e.g., melasma, freckles, and post-inflammatory hyperpigmentation) and hypopigmentation (e.g., vitiligo). Although these conditions are not life-threatening, they can significantly impact patients’ self-esteem and mental health, potentially leading to depression and a reduced quality of life. Studies have indicated that effective treatment of pigmentation disorders can improve patients’ psychological well-being and overall quality of life. However, current clinical treatments exhibit limited efficacy, with high recurrence rates and potential side effects, underscoring the need for improved therapeutic approaches. Additionally, the complex pathophysiological mechanisms underlying pigmentation disorders remain incompletely understood, posing further challenges to the development of novel treatments [1,2,3].

Abnormal pigmentation is primarily attributed to dysregulation in the melanogenesis process. Melanin is synthesized in melanocytes, which are located in the basal layer of the epidermis, and its production is regulated by external factors, such as ultraviolet (UV) radiation, and internal factors, such as hormonal changes. Disruption of melanogenic homeostasis by these factors can result in temporary or permanent pigmentation changes, leading to hyperpigmentation or hypopigmentation disorders. Melanin synthesis occurs within specialized organelles known as melanosomes and involves key melanogenic enzymes, such as tyrosinase, tyrosinase-related protein 1 (TRP-1), and tyrosinase-related protein 2 (TRP-2). Tyrosinase, a pivotal enzyme in the melanin biosynthetic pathway, catalyzes the hydroxylation of tyrosine to 3,4-dihydroxyphenylalanine (DOPA) and the subsequent oxidation of DOPA to DOPA quinone. TRP-1 and TRP-2 regulate downstream stages of melanin synthesis [4,5,6].

Microphthalmia-associated transcription factor (MITF) is a master regulator of melanocyte-specific gene expression, controlling the transcription of tyrosinase, TRP-1, and TRP-2. MITF also plays a critical role in melanocyte survival, proliferation, differentiation, and melanin production. Melanogenesis is modulated by various signaling pathways, including the PKA/cAMP, Wnt/β-catenin, MAPK/ERK, and PI3K/AKT pathways. The PKA/cAMP pathway is activated by α-melanocyte-stimulating hormone (α-MSH), which binds to the melanocortin 1 receptor (MC1R), increasing intracellular cAMP levels. This activates protein kinase A (PKA), which phosphorylates the cAMP response element-binding protein (CREB), inducing MITF activation and promoting melanin synthesis. The Wnt/β-catenin pathway stabilizes β-catenin, which translocates to the nucleus and enhances MITF expression, supporting melanocyte survival and differentiation. The MAPK/ERK pathway, activated by the KIT receptor, modulates MITF activity through phosphorylation, thereby regulating the expression of melanogenic enzymes. The PI3K/AKT pathway enhances melanocyte survival and resilience to oxidative stress by activating AKT, further supporting cell survival [7,8].

While melanin production plays a critical role in protecting the skin from harmful UV radiation, defects in melanocyte function can lead to melanin loss, resulting in hypopigmentation disorders such as vitiligo. Vitiligo is characterized by the destruction of melanocytes in affected areas, and oxidative stress is considered a key pathogenic factor in melanocyte depletion. Current treatments for vitiligo include narrow-band ultraviolet B (NB-UVB) therapy, topical corticosteroids, and oral medications. However, these therapies often exhibit limited efficacy and are associated with adverse effects. Given these limitations, natural compounds that stimulate melanogenesis have garnered attention as potential therapeutic agents for improving hypopigmentation disorders. Research on natural products provides a foundation for developing safer and more effective treatments for pigmentation disorders by addressing the drawbacks of existing therapies [9,10,11]. Various natural compounds have been identified as activators that enhance the activity of tyrosinase, showing potential therapeutic effects in the treatment of vitiligo. These include psoralen, quercetin, coumarin derivatives, and other bioactive compounds [12,13,14].

**Figure 1:**
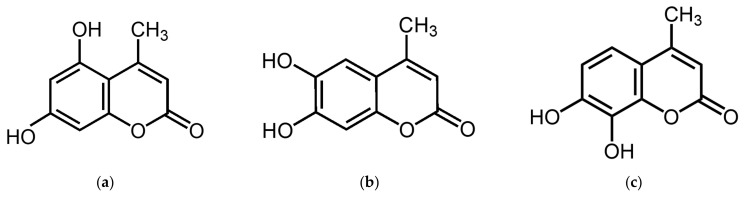
The chemical structures of 5,7-dihydroxy-4-methylcoumarin (**a**), 6,7-dihydroxy-4-methylcoumarin (**b**), and 7,8-dihydroxy-4-methylcoumarin (**c**).

5,7-Dihydroxy-4-methylcoumarin (5,7D-4MC) is a member of the coumarin family and possesses a benzopyrone core structure (1,2-benzopyrone or chromen-2-one). This structure consists of a benzene ring fused with a lactone ring (α-pyrone), with a methyl group (-CH₃) substituted at the 4-position of the lactone ring, classifying it as a “4-methyl coumarin” (Figure 1a). 5,7D-4MC has been identified as a naturally occurring coumarin compound isolated from various plant sources, including *Eranthis longistipitata* and Mexican tarragon (*Tagetes lucida*). Pharmacologically, 5,7D-4MC exhibits a wide range of bioactivities, including antioxidant, anti-inflammatory, and antiplatelet effects. It has been shown to selectively inhibit cyclooxygenase-1 (COX-1) and act as a competitive antagonist of thromboxane A_2_ (TXA_2_) receptors. Additionally, its ability to mitigate oxidative stress-related damage highlights its therapeutic potential for the prevention and treatment of inflammatory disorders, cardiovascular diseases, and drug-induced ototoxicity [15,16,17,18,19]. To date, no studies have reported the effects of 5,7D-4MC on melanogenesis or its potential applications in skin-related disorders. Therefore, this study aimed to investigate the stimulatory effects of 5,7D-4MC on melanogenesis in B16F10 murine melanoma cells. The underlying mechanism of this effect was evaluated by analyzing the expression of the MITF transcription factor and key phosphorylation regulators associated with the PKA, PI3K/AKT, and GSK-3β signaling pathways. Additionally, to ensure its suitability for topical application, a human primary skin irritation test was conducted to evaluate the dermatological safety profile of 5,7D-4MC.

## 2. Results

### 2.1. Effects of 5,7D-4MC on the Melanin Production

**Figure 2:**
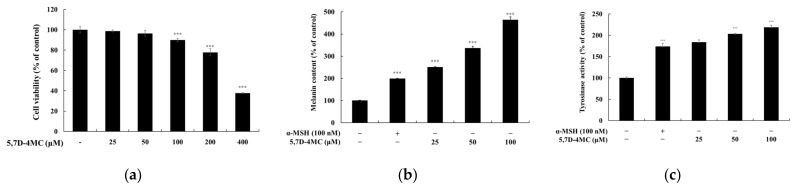
B16F10 melanoma cells were treated with 5,7D-4MC at various concentrations (12.5 to 400 µM) for 72 h, and cell viability (**a**), melanin production (**b**), and tyrosinase activity (**c**) were assessed. Cell viability was measured using the MTT assay and expressed as a percentage relative to untreated cells. To evaluate melanogenesis, α-MSH-stimulated cells were treated with 5,7D-4MC at 12.5, 25, and 50 µM, with α-MSH (100 nM) serving as a positive control in both melanin production and tyrosinase activity assays. In all experiments, results are expressed as the mean ± SD from three independent replicates. Statistical significance compared to the unstimulated control group is indicated as * *p* < 0.05, ** *p* < 0.01, *** *p* < 0.001.

To determine the safe concentrations of 5,7D-4MC, its cytotoxicity was evaluated in B16F10 melanoma cells using the 3-(4,5-dimethylthiazol-2-yl)-2,5-diphenyltetrazolium bromide (MTT) assay. The cells were treated with 5,7D-4MC at concentrations ranging from 25 to 400 μM. As shown in Figure 2a, no apparent cytotoxicity was observed in B16F10 cells at concentrations of 25, 50, and 100 μM. Even at the highest concentration of 100 μM, cell viability remained above 90%. Therefore, we conclude that 5,7D-4MC is not toxic to B16F10 cells at concentrations below 100 μM. Subsequent experiments were conducted using 5,7D-4MC at concentrations of 25, 50, and 100 μM, all of which showed no cytotoxic effects. Next, we examined the effect of 5,7D-4MC on melanin synthesis by measuring the melanin content in the cells after incubation with 5,7D-4MC at different concentrations (25, 50, and 100 μM) for 72 h. Alpha-melanocyte-stimulating hormone (α-MSH), a major factor that stimulates melanin synthesis in the skin, was used as a positive control. As shown in Figure 2b, melanin content in the positive control group increased by approximately 198.0% compared to the untreated control group. In comparison, the 5,7D-4MC-treated groups (25, 50, and 100 μM) showed increases in melanin content of 250.1%, 336.0%, and 463.0%, respectively. These results demonstrate that melanin levels were significantly increased by 5,7D-4MC in a concentration-dependent manner, indicating that 5,7D-4MC stimulates melanogenesis in B16F10 cells without causing cytotoxicity.

### 2.2. Effects of 5,7D-4MC on the Intracellular Tyrosinase Activity

Tyrosinase activity was analyzed to investigate the possible underlying mechanisms associated with 5,7D-4MC-induced stimulation of melanin synthesis in B16F10 cells. Tyrosinase is a rate-limiting enzyme involved in the oxidation of tyrosine and 3,4-dihydroxy-L-phenylalanine (L-DOPA) during the initial stages of melanin synthesis. After incubating B16F10 cells with 5,7D-4MC at different concentrations (25, 50, and 100 μM) for 72 h, intracellular tyrosinase activity was measured. As shown in Figure 2c, 5,7D-4MC significantly increased intracellular tyrosinase activity in a concentration-dependent manner. Compared to the untreated control group, the tyrosinase activity in the positive control group increased by 174.0%, whereas the tyrosinase activity in the 5,7D-4MC-treated groups (25, 50, and 100 μM) increased by 183.8%, 203.1%, and 218.6%, respectively. Notably, treatment with 100 μM 5,7D-4MC resulted in tyrosinase activity that was approximately 25.6% higher than that in the positive control group.

### 2.3. Molecular Docking Analysis

**Figure 3:**
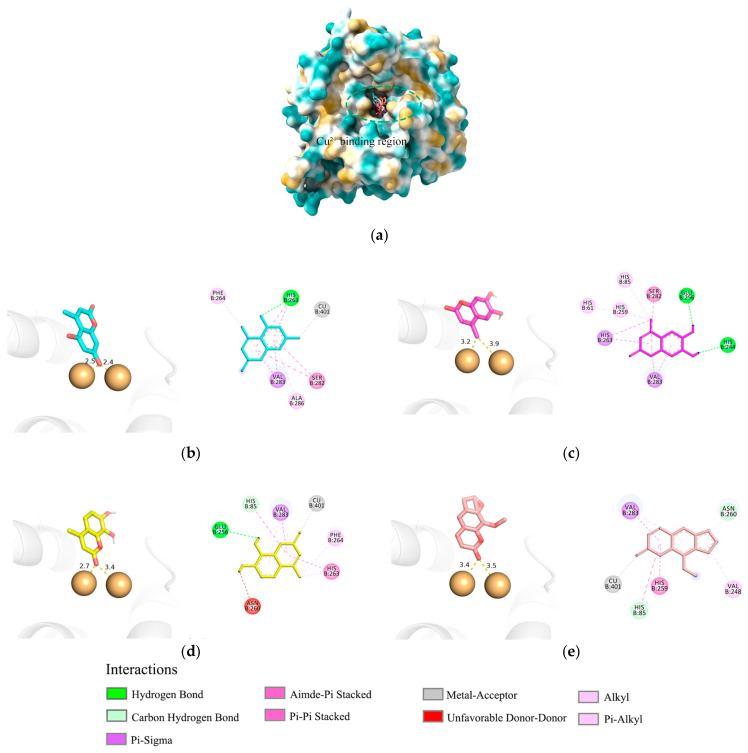
Molecular docking analysis of mTYR–ligand complexes. (**a**) Copper ion binding region of mushroom tyrosinase, (**b**) Binding interaction of mTYR-5,7D-4MC complex, (**c**) Binding interaction of mTYR-6,7D-4MC complex, (**d**) Binding interaction of mTYR-7,8D-4MC complex, (**e**) Binding interaction of mTYR-8-MOP complex.

To further investigate the mechanism of tyrosinase activation, this study utilized mushroom tyrosinase (PDB ID: 2Y9X) as a model and focused on 5,7D-4MC, 6,7-dihydroxy-4-methylcoumarin (6,7D-4MC), and 7,8-dihydroxy-4-methylcoumarin (7,8D-4MC), with 8-methoxypsoralen (8-MOP), a psoralen-derived tyrosinase activator, selected as the control ligand for molecular docking studies (Figure 3a). The molecular docking results of 5,7D-4MC (Figure 3b), 6,7D-4MC (Figure 3c), 7,8D-4MC (Figure 3d), and 8-MOP (Figure 3e) revealed binding energies of −6.8, −7.0, −7.3, and −6.8 kcal/mol, respectively. The three coumarin derivatives (5,7D-4MC, 6,7D-4MC, and 7,8D-4MC) exhibited binding energies similar to that of the activator 8-MOP, suggesting that they may have strong binding affinity with tyrosinase. Although 6,7D-4MC and 7,8D-4MC demonstrated strong binding affinity in molecular docking, previous studies have shown that they do not exhibit activation of tyrosinase [20]. This suggests that these compounds may interact with tyrosinase through a different mechanism, lacking the direct activation of enzyme activity.

During the catalytic process of mushroom tyrosinase, the copper ion (Cu^2+^) is essential for substrate oxidation and maintaining the enzyme’s structural stability [21]. Previous studies have suggested that the binding of the phenolic group in synthetic coumarin derivatives to copper ions, similar to that observed with 8-MOP, may be a key factor contributing to their potential as enzyme activators [22]. The molecular docking results indicated that in the final optimal conformation of 5,7D-4MC, the hydroxyl group of the coumarin ring is closer to the Cu^2+^ compared to that of 6,7D-4MC and 7,8D-4MC, which may result in a stronger interaction with tyrosinase and more effectively promote enzyme activity. Although 5,7D-4MC (hydroxyl), 7,8D-4MC (carbonyl), and the activator 8-MOP (carbonyl) all formed favorable metal–receptor interactions with the Cu^2+^, the phenolic group of 7,8D-4MC forms an unfavorable donor–donor interaction with the amino acid residue ASN-260 of tyrosinase, which may contribute to its lower activity. On the other hand, the non-polar methyl group of 6,7D-4MC, despite being relatively close to the Cu^2+^, does not form a coordination bond with the Cu^2+^ due to its lack of lone pair electrons, which may be a key factor affecting its activation potential. Overall, for 5,7D-4MC and its derivatives, the formation of coordination bonds with the copper ion and a closer proximity to the copper may be more favorable for promoting tyrosinase catalytic activity. These findings provide theoretical insights into the design of new tyrosinase activators.

### 2.4. Molecular Dynamics (MD) Simulations Analysis

**Figure 4:**
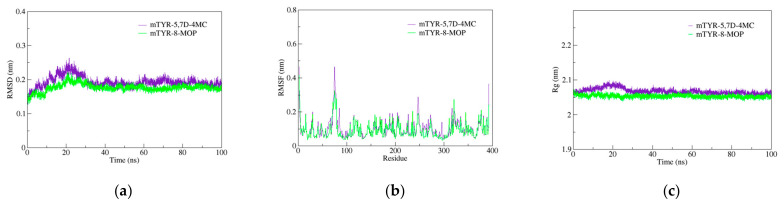

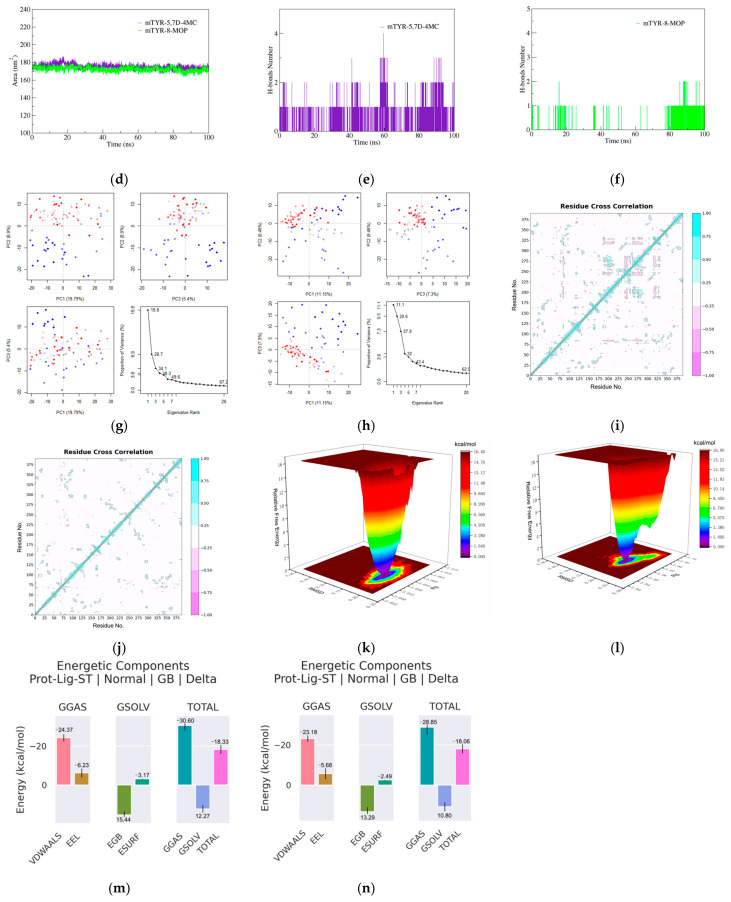
Molecular dynamics simulation analysis of mTYR–ligand complexes. (**a**) RMSD plot of mTYR–ligand complexes, (**b**) RMSF plot of mTYR–ligand complexes, (**c**) Rg plot of mTYR–ligand complexes, (**d**) SASA plot of mTYR–ligand complexes, (**e**) H-bonds analysis of mTYR-5,7D-4MC complex, (**f**) H-bonds analysis of mTYR-8-MOP complex, (**g**) PCA of mTYR-5,7D-4MC complex, (**h**) PCA of mTYR-8-MOP complex, (**i**) DCCM analysis of mTYR-5,7D-4MC complex, (**j**) DCCM analysis of mTYR-8-MOP complex, (**k**) Gibbs free energy landscape of mTYR-5,7D-4MC complex, (**l**) Gibbs free energy landscape of mTYR-8-MOP complex, (**m**) Binding free energy of mTYR-5,7D-4MC complex, (**n**) Binding free energy of mTYR-8-MOP complex.

Based on molecular docking analysis, 5,7D-4MC and 8-MOP exhibit similar binding modes within mTYR. MD simulations were subsequently performed to compare the stability and binding strength of the two complexes. In terms of RMSD, both complexes reached a relatively stable state at approximately 30 ns, with RMSD values stabilizing at 0.20 ± 0.05 nm (Figure 4a). RMSF analysis identified the flexible regions primarily within the 70–80 residue range, with fluctuation amplitudes below 0.5 nm, indicating that ligand binding did not induce significant structural perturbations in the protein (Figure 4b). Moreover, analyses of the radius of gyration (Rg, Figure 4c) and solvent-accessible surface area (SASA, Figure 4d) showed nearly overlapping curves for both complexes, reflecting comparable thermodynamic stability and conformational dynamics. Regarding the hydrogen bond analysis, the mTYR-5,7D-4MC complex consistently maintained a single stable hydrogen bond throughout the simulation (Figure 4e), whereas the mTYR-8-MOP complex displayed relatively stable hydrogen bonding only during the 80–100 ns interval (Figure 4f). Principal component analysis (PCA) revealed that the first three principal components accounted for 34.1% and 27.9% of the total variance in the mTYR-5,7D-4MC and mTYR-8-MOP complexes, respectively (Figure 4g,h). The axes represent the protein’s conformational space, with each dot showing a configuration. The blue-to-red color gradient indicates the simulation’s progression, from start (blue) to end (red). The slightly higher variance observed for the 5,7D-4MC complex suggests more notable conformational changes. Dynamic cross-correlation matrix (DCCM) analysis further highlighted significant differences in correlated motions between the two complexes. The mTYR-5,7D-4MC complex exhibited more significant positive (light blue) and negative (pink) correlated motions in the 200–350 residue region (Figure 4i), whereas the mTYR-8-MOP complex (Figure 4j) displayed a higher degree of conformational stability.

In terms of ligand-target binding strength, Gibbs free energy landscape (FEL) analysis revealed that both mTYR-5,7D-4MC and mTYR-8-MOP complexes formed a single energy cluster, indicative of strong binding strength (Figure 4k,l). Binding free energy calculations using the MMPBSA method over the last 20 ns of stable trajectories yielded values of −18.33 kcal/mol for the mTYR-5,7D-4MC complex (Figure 4m) and −18.06 kcal/mol for the mTYR-8-MOP complex (Figure 4n), demonstrating strong interactions in both cases. In summary, MD simulations revealed that 5,7D-4MC and 8-MOP share similar binding modes and thermodynamic stability within mTYR, both exhibiting strong binding affinities.

### 2.5. Effects of 5,7D-4MC on the Expression of Melanogenic Enzymes

**Figure 5:**
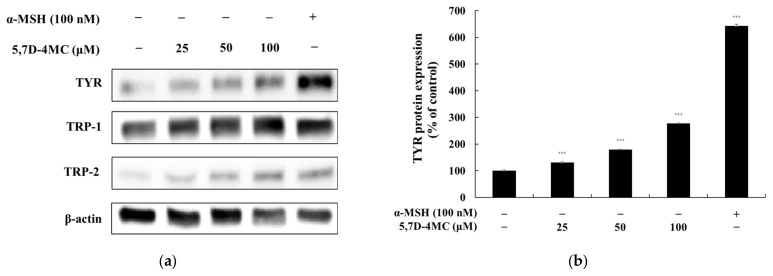

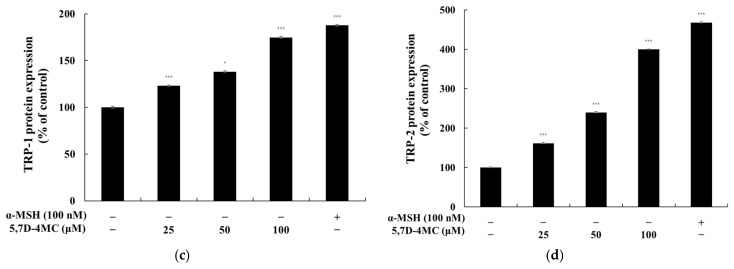
The effect of 5,7D-4MC on tyrosinase, TRP-1, and TRP-2 protein expression in α-MSH-stimulated B16F10 cells. The expression levels of tyrosinase, TRP-1, and TRP-2 were determined by Western blot analysis. (**a**) Western blot results, (**b**) tyrosinase/β-actin protein expression, (**c**) TRP-1/β-actin protein expression, (**d**) TRP-2/β-actin protein expression: α-MSH (100 nM) was used as a positive control. The relative intensity of the protein bands was quantified using ImageJ2 software, and the values were normalized to the corresponding loading control. Untreated cells were set as 100%. Data are presented as the mean ± SD of at least three independent experiments. * *p* < 0.05, ** *p* < 0.01, *** *p* < 0.001 compared to the α-MSH alone group.

Since 5,7D-4MC treatment increased tyrosinase activity, the protein expression level of tyrosinase in B16F10 cells was evaluated using Western blotting. At 72 h after treatment with 5,7D-4MC, tyrosinase expression was significantly increased in a concentration-dependent manner. Tyrosinase-related proteins TRP-1 and TRP-2 also play important roles in regulating melanin synthesis; therefore, we examined the expression of TRP-1 and TRP-2 using Western blotting. As shown in Figure 5, 5,7D-4MC treatment led to an increase in the expression of both proteins. In particular, after treatment with 100 μM 5,7D-4MC, tyrosinase, TRP-1, and TRP-2 protein expression levels increased by approximately 177.0%, 74.7%, and 299.6%, respectively, compared to the untreated control. Notably, after treatment with 100 μM 5,7D-4MC, the expression levels of TRP-1 and TRP-2 increased to a level nearly comparable to that of the 100 nM control group. Taken together, these results indicate that 5,7D-4MC stimulates melanogenesis by positively regulating the expression of melanogenic proteins.

**Figure 6:**
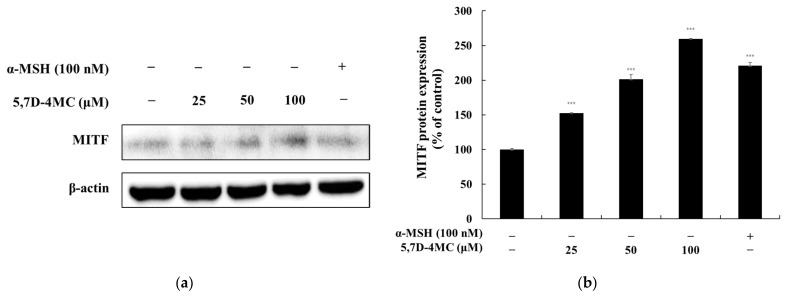
The effect of 5,7D-4MC on MITF protein expression in α-MSH-stimulated B16F10 cells. (**a**) Western blot results, (**b**) MITF/β-actin protein expression. α-MSH (100 nM) was used as a positive control. The relative intensity of the protein bands was quantified using ImageJ software, and the values were normalized to the corresponding loading control. Untreated cells were set as 100%. Data are presented as the mean ± SD of at least three independent experiments. * *p* < 0.05, ** *p* < 0.01, *** *p* < 0.001 compared to the α-MSH alone group.

MITF is a key regulator of melanogenesis, controlling the expression of melanogenic genes such as tyrosinase, TRP-1, and TRP-2 by binding to the M-box motif in their promoter regions [4,5,6]. To further elucidate the role of MITF in 5,7D-4MC-induced melanogenesis, we examined its protein expression following treatment. As shown in Figure 6, MITF expression levels in B16F10 cells increased in a concentration-dependent manner after exposure to 5,7D-4MC. Notably, at a concentration of 100 μM, MITF protein expression was approximately 159.7% higher than that of the untreated control group. These findings indicate that MITF plays a critical role in mediating the melanogenic effects of 5,7D-4MC.

### 2.6. Effects of 5,7D-4MC on the PKA Signaling Pathway

**Figure 7:**
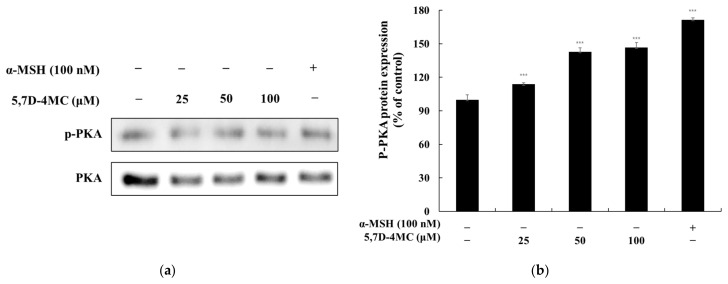
The effect of 5,7D-4MC on PKA protein expression in α-MSH-stimulated B16F10 cells. PKA expression was determined by Western blot analysis. (**a**) Western blot results, (**b**) p-PKA/PKA protein expression. α-MSH (100 nM) was used as a positive control. The relative intensity of the protein bands was quantified using ImageJ software, and the values were normalized to the corresponding loading control. Untreated cells were set as 100%. Data are presented as the mean ± SD of at least three independent experiments. * *p* < 0.05, ** *p* < 0.01, *** *p* < 0.001 compared to the α-MSH alone group.

α-MSH binds to the melanocortin 1 receptor (MC1R), triggering an increase in intracellular cyclic adenosine monophosphate (cAMP) levels. Elevated cAMP activates protein kinase A (PKA), which subsequently phosphorylates and activates the cAMP response element-binding protein (CREB). Activated CREB enhances the expression of MITF, leading to the transcriptional activation of tyrosinase, TRP-1, and TRP-2—key genes involved in melanin synthesis. This cascade ultimately enhances melanin production, regulating cellular pigment synthesis [23,24]. To determine whether 5,7D-4MC influences the cAMP-PKA signaling pathway in B16F10 cells, we performed Western blot analysis to assess its effect on PKA phosphorylation. As shown in Figure 7, treatment with 5,7D-4MC at all tested concentrations led to a significant increase in PKA expression, exceeding 46.8% compared to the untreated control group. These findings suggest that 5,7D-4MC promotes melanogenesis through activation of the cAMP-PKA signaling pathway.

### 2.7. Effects of 5,7D-4MC on the GSK3β Signaling Pathway

**Figure 8:**
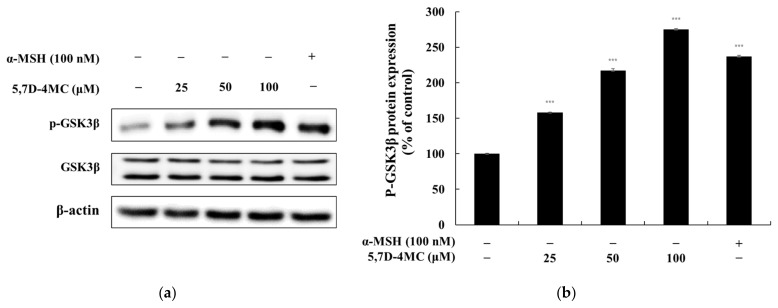
The effect of 5,7D-4MC on GSK3β protein expression in α-MSH-stimulated B16F10 cells. GSK3β expression was determined by Western blot analysis. (**a**) Western blot results, (**b**) p-GSK3β/GSK3β protein expression. α-MSH (100 nM) was used as a positive control. The relative intensity of the protein bands was quantified using ImageJ software, and the values were normalized to the corresponding loading control. Untreated cells were set as 100%. Data are presented as the mean ± SD of at least three independent experiments. * *p* < 0.05, ** *p* < 0.01, *** *p* < 0.001 compared to the α-MSH alone group.

Previous studies have demonstrated the pivotal role of GSK3β in regulating melanogenesis via the Wnt/β-catenin signaling pathway. When Wnt ligands bind to their receptors, GSK3β is inactivated, preventing the phosphorylation and subsequent degradation of β-catenin. This allows β-catenin to accumulate in the cytoplasm and translocate into the nucleus, where it stimulates MITF expression. MITF, in turn, enhances the transcription of key melanogenic genes, including tyrosinase, TRP-1, and TRP-2, thereby promoting melanin synthesis. To determine whether 5,7D-4MC influences GSK3β activity, we examined its effect on GSK3β phosphorylation levels. As shown in Figure 8, treatment with 5,7D-4MC resulted in a concentration-dependent increase in GSK3β phosphorylation. These findings suggest that 5,7D-4MC enhances pigmentation by upregulating MITF expression through the GSK3β signaling pathway [25].

### 2.8. Effects of 5,7D-4MC on the PI3K/AKT Signaling Pathways

**Figure 9:**
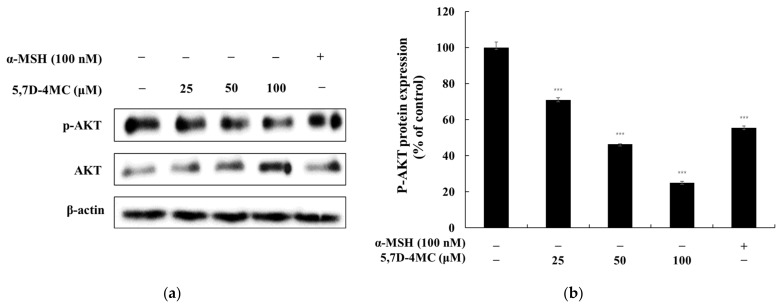
The effect of 5,7D-4MC on AKT protein expression in α-MSH-stimulated B16F10 cells. AKT expression was determined by Western blot analysis. (**a**) Western blot results, (**b**) p-AKT/AKT protein expression. α-MSH (100 nM) was used as a positive control. The relative intensity of the protein bands was quantified using ImageJ software, and the values were normalized to the corresponding loading control. Untreated cells were set as 100%. Data are presented as the mean ± SD of at least three independent experiments. * *p* < 0.05, ** *p* < 0.01, *** *p* < 0.001 compared to the α-MSH alone group.

Previous studies have shown that the PI3K/AKT signaling pathway negatively regulates melanogenesis in melanocytes and melanoma cells [26,27]. To determine whether 5,7D-4MC affects this pathway in B16F10 cells, we performed Western blot analysis to assess its impact on ERK and AKT phosphorylation. As shown in Figure 9, treatment with 5,7D-4MC led to a reduction in AKT phosphorylation levels. Notably, at a concentration of 100 μM, AKT phosphorylation decreased by 74.9% compared to the untreated control group. These findings suggest that 5,7D-4MC modulates the PI3K/AKT signaling pathway in B16F10 cells, which may contribute to the upregulation of melanogenesis.

### 2.9. Safety of 5,7D-4MC for Human Skin

Human skin requires protection from environmental stressors and chemical exposure in cosmetics and pharmaceuticals, particularly in vulnerable populations such as children. Therefore, it is essential to evaluate the potential of cosmetic ingredients to induce acute skin irritation. A skin irritation assay was performed to investigate the effects of the topical application of 5,7D-4MC at concentrations of 50 μM and 100 μM, dissolved in squalene. In accordance with PCPC guidelines and Dermapro Ltd. protocols, 32 women (mean age 43.03 ± 5.17 years) participated in a patch test in which 20 μL of 5,7D-4MC was applied to a cleansed area on their backs for 24 h. Dermatological assessments were conducted 20 min and 24 h post-patch removal. The findings demonstrated that 5,7D-4MC exhibits hypoallergenic characteristics with respect to primary skin irritation (Table 1).

## 3. Discussion

Melanin is an essential pigment that protects the skin from ultraviolet (UV) radiation and plays a key role in thermoregulation. Deficiencies in melanocyte function are linked to hypopigmentation disorders, such as vitiligo and albinism. Vitiligo, in particular, is characterized by the progressive loss of melanocytes, leading to localized or widespread depigmentation. This condition not only affects the patient’s appearance but can also cause psychological distress and negatively impact their quality of life [1,2,3]. Currently, vitiligo treatment options include narrow-band UVB (NB-UVB) therapy, corticosteroids, and immunomodulators. However, these approaches have limited effectiveness, potential adverse effects, and a high recurrence rate, highlighting the need for safer and more effective therapeutic strategies. Since the precise pathophysiology of vitiligo remains incompletely understood, natural compounds capable of enhancing melanin synthesis with minimal side effects have gained attention as promising treatment candidates. Developing novel, safe, and effective therapeutics for vitiligo is crucial not only for improving patient well-being but also for advancing dermatological research [9,10,11].

5,7-Dihydroxy-4-Methylcoumarin (5,7D-4MC) has been reported to exhibit various biological activities, including anti-inflammatory, antiplatelet, and cytoprotective effects, making it a promising candidate for therapeutic applications [12,13,14,15,16]. However, its involvement in melanin synthesis remains insufficiently explored. In this study, we investigated the impact of 5,7D-4MC on melanin synthesis in B16F10 murine melanoma cells and observed that the compound, even at concentrations up to 100 µM, did not exhibit cytotoxic effects (maintaining over 90% cell viability) while significantly increasing melanin production and tyrosinase activity. Additionally, Western blot analysis demonstrated that 5,7D-4MC upregulated the expression of melanin synthesis-related proteins, including MITF, tyrosinase, TRP-1, and TRP-2, and activated key melanin biosynthesis signaling pathways such as PI3K/AKT, PKA, and Wnt/β-catenin. The ability of 5,7D-4MC to enhance melanin synthesis is structurally noteworthy. As a coumarin derivative bearing two hydroxyl (-OH) groups, its antioxidant properties have been documented in studies on phenolic compounds and flavonoid ring structures. These studies suggest that hydroxyl groups contribute to antioxidant activity by donating electrons and scavenging free radicals. Typically, antioxidant effects are associated with the inhibition of melanogenesis, as the process heavily depends on oxidative reactions. Antioxidants can suppress melanin synthesis by eliminating reactive oxygen species (ROS), inhibiting tyrosinase, and reducing dopaquinone levels [28,29]. However, in contrast to this general mechanism, 5,7D-4MC was observed to enhance melanin synthesis.

Our previous studies have explored the relationship between melanin synthesis and various classes of compounds, including flavonoids, coumarins, chalcones, and psoralen derivatives. Generally, compounds possessing two or more hydroxyl (-OH) groups tend to either suppress melanin synthesis or exhibit negligible effects at concentrations that do not compromise cell viability. Notably, compounds that enhance melanin production frequently contain methoxy (-OCH₃) functional groups [30,31,32,33]. Recent investigations have revealed that structurally related analogs, such as 6,7-dihydroxy-4-methylcoumarin (6,7D-4MC) and 7,8-dihydroxy-4-methylcoumarin (7,8D-4MC), do not significantly affect melanin synthesis under identical experimental conditions (Figure 1) [20].

Several hypotheses could account for these differences. First, the position of hydroxyl groups in coumarin derivatives may be crucial in determining their role in either inhibiting or activating tyrosinase. In the case of 5,7D-4MC, the hydroxyl groups at the fifth and seventh positions of the benzene ring might facilitate enzyme activation by donating electrons during interactions with melanosomal enzymes, particularly tyrosinase. In contrast, 6,7D-4MC and 7,8D-4MC possess an ortho-dihydroxy configuration, which enhances antioxidant activity but may negatively impact enzyme activation [34]. Previous studies indicate that 6-hydroxycoumarin and 7-hydroxycoumarin serve as weak tyrosinase substrates, whereas 3-hydroxycoumarin functions as an effective tyrosinase inhibitor, and 4-hydroxycoumarin lacks inhibitory effects [35]. The absence of a hydroxyl group at position 3 might explain the weak inhibitory potential of these compounds. However, the presence of hydroxyl groups at positions 6 or 7 suggests they could act as weak substrates or activators of tyrosinase. Second, differences in resonance structure stability may contribute to the observed effects [36]. The 5,7-dihydroxy arrangement can adopt a quinone-like resonance form that may promote enzyme activation required for melanin synthesis. In contrast, the 6,7- and 7,8-dihydroxy structures exhibit greater resonance stability, making them effective in scavenging free radicals but potentially interfering with the redox reactions necessary for melanin synthesis. Third, variations in interactions with the allosteric site of tyrosinase could play a role. The allosteric site modulates enzyme function through conformational changes upon ligand binding [37]. 5,7D-4MC may engage with this site to regulate enzyme activity, whereas 6,7D- and 7,8D-4MC might either fail to bind effectively or bind without inducing functional alterations. Fourth, interactions with copper ions (Cu^2+^) are essential. As a key cofactor, copper is required to maintain the structural integrity of tyrosinase and enhance its substrate-binding capacity [38].

The molecular docking study on the activation mechanism of tyrosinase revealed that both 5,7D-4MC and 8-MOP interact with the copper ion in a similar manner. During the MD simulations, the mTYR-5,7D-4MC complex exhibited stability comparable to that of the mTYR-8-MOP complex, as reflected by the RMSD, RMSF, Rg, SASA, and h-bonds analyses. PCA and DCCM further showed similar dynamic behavior between the two complexes. In terms of binding affinity, both complexes formed stable single-energy clusters, as determined by Gibbs free energy analysis. The binding free energies of the mTYR-5,7D-4MC and mTYR-8-MOP complexes were −18.33 kcal/mol and −18.06 kcal/mol, respectively. In conclusion, 5,7D-4MC, as a tyrosinase activator, demonstrated binding stability and affinity similar to 8-MOP in the in silico analysis, providing a promising candidate for the development of coumarin-based activators. Fifth, there may be differences in intracellular signaling pathways. 5,7D-4MC activates pathways such as MAPK/ERK, PKA/cAMP, and Wnt/β-catenin, which promote melanin synthesis. However, 6,7D- and 7,8D-4MC may not activate these pathways or may even induce inhibitory signals. Lastly, differences in the hydrophobicity of 4-methylcoumarin derivatives may influence their activity. Although all 4-methylcoumarin derivatives contain a methyl group (-CH₃) at the fourth position, the arrangement of hydroxyl groups affects the hydrophilic–lipophilic balance. 5,7D-4MC exhibits higher lipophilicity, facilitating its access to the cell membrane and enzyme binding sites [39], whereas 6,7D- and 7,8D-4MC may exhibit increased hydrophilicity, limiting their accessibility to target sites. In summary, the ability of 5,7D-4MC to enhance melanin synthesis may be attributed to the strategic placement of its hydroxyl groups, which optimize interactions with melanin-related enzymes and activate melanogenic signaling pathways. Conversely, 6,7D- and 7,8D-4MC are unlikely to contribute to melanin production due to their structural characteristics, which may inhibit enzyme activation or fail to engage in the necessary redox interactions.

The unique structure and melanin-promoting effect of 5,7D-4MC hold significant scientific and industrial implications. As a synthetic precursor, 5,7D-4MC’s phenolic hydroxyl groups can undergo chemical modifications such as esterification, alkylation, and acetylation [40]. Additionally, the hydroxyl groups at the fifth and seventh positions enable electron density modulation and hydrogen bonding, making 5,7D-4MC applicable in studies involving enzyme reactions and ligand–receptor interactions. Thus, 5,7D-4MC may serve as a valuable chemical probe for investigating melanogenesis mechanisms. From an industrial perspective, drug development based on 5,7D-4MC could be applied to the treatment of vitiligo and other hypopigmentation disorders. Additionally, it could be utilized as an active ingredient in functional cosmetics aimed at improving skin tone and maintaining pigment balance. Structure–activity relationship (SAR) studies could also facilitate the design of derivatives to optimize efficacy.

The findings of this study suggest that 5,7D-4MC has the potential to be an effective agent for addressing hypopigmentation disorders. Its ability to enhance melanin synthesis positions it as a promising candidate for cosmetic applications and the treatment of pigment-related conditions such as vitiligo. To explore its safety profile, a skin irritation test was conducted with 32 participants in accordance with OECD guidelines. As summarized in Table 1, 5,7D-4MC exhibited no adverse skin reactions at concentrations of 50 µM and 100 µM, indicating a favorable safety profile. However, this study has certain limitations. The experiments were conducted using the B16F10 murine melanoma cell line, which is widely used in melanin-related research. While 5,7D-4MC effectively stimulated melanin synthesis at the cellular level, these results may not be directly translatable to human melanocytes or clinical conditions. Therefore, further investigations involving human melanocytes and clinical trials will be necessary to fully establish the therapeutic potential of 5,7D-4MC and support its practical application in dermatology and cosmetics.

## 4. Materials and Methods

### 4.1. Chemicals and Reagents

5,7-Dihydroxy-4-methylcoumarin (5,7D-4MC) was purchased from Tokyo Chemical Industry (Chuo-ku, Japan). Dulbecco’s Modified Eagle’s Medium (DMEM), penicillin–streptomycin (10,000 U/mL), fetal bovine serum (FBS), a BCA protein assay kit used for protein quantification, and a hydrophilic polyvinylidene fluoride membrane filter used for Western blotting were obtained from Thermo Fisher Scientific (Waltham, MA, USA). (4,5-dimethylthiazol-2-yl)-2,5-diphenyltetrazolium bromide (MTT), a protease inhibitor cocktail used for cell lysis; α-melanocyte-stimulating hormone (α-MSH, used as a control); sodium hydroxide (NaOH); sodium phosphate monobasic; sodium phosphate dibasic; and 2-mercaptoethanol were purchased from Sigma-Aldrich (St. Louis, MO, USA).

The 2× Laemmli sample buffer and 10% Tween 20 used for Western blot sample preparation were purchased from Bio-Rad (Hercules, CA, USA). Skim milk was purchased from BD Difco (Sparks, MD, USA), and bovine serum albumin (BSA) was obtained from Bovostar (Bovogen, Melbourne, Australia). Dimethyl sulfoxide (DMSO), radioimmunoprecipitation assay (RIPA) buffer, 20× TBS buffer (pH 7.6) used to prepare 1× TBST, 10× Tris-glycine buffer, and phosphate-buffered saline (PBS) were purchased from Biosesang (Seongnam, Republic of Korea). Primary antibodies, including tyrosinase (SC-20035), TRP-1 (SC-166857), TRP-2 (SC-74439), Actin-β antibody (C4) (C-47778_s), and MITF (SC-71588), were purchased from Santa Cruz Biotechnology (Dallas, TX, USA). p-PKA (5661S), PKA (4782S), p-GSK-3β (9322S), GSK-3β (5676S), p-AKT (9271S), AKT (9272S), anti-rabbit IgG HRP-linked antibody (7074S), and anti-mouse IgG HRP-linked antibody (7076S) were obtained from Cell Signaling Technology (Danvers, MA, USA).

### 4.2. Cell Culture

In this experiment, B16F10 mouse melanoma cells were obtained from ATCC (The Global Bioresource Center, Manassas, VA, USA). The cells were cultured in Dulbecco’s Modified Eagle Medium (DMEM, Gibco, Waltham, MA, USA) supplemented with 10% fetal bovine serum (FBS, Gibco, Waltham, MA, USA) and 1% penicillin–streptomycin (P/S, Gibco, Waltham, MA, USA). The culture conditions were maintained at 37 °C in a humidified atmosphere containing 5% CO_2_ using a CO_2_ incubator (Thermo Fisher Scientific, Waltham, MA, USA). The culture medium was replaced every 48 h, and cells were subcultured upon reaching 80–90% confluency using 0.25% trypsin-EDTA (Gibco, Waltham, MA, USA). For experimental treatments, cells were seeded in 6-well plates at a density of 2 × 10^5^ cells per well and allowed to adhere overnight before compound treatment. The final concentrations of 5,7-Dihydroxy-4-Methylcoumarin and control substances were prepared by diluting stock solutions in fresh DMEM media. All experiments were conducted in triplicate to ensure reproducibility.

### 4.3. Measurement of Cell Viability

B16F10 cells were seeded at a density of 1.5 × 10^4^ cells/well in a 24-well plate and incubated for 24 h. Afterward, the samples were treated and further incubated for 72 h at 37 °C in a 5% CO_2_ incubator. Following the reaction, all media from each well were removed, and 500 μL of MTT reagent at a concentration of 0.2 mg/mL was added to each well. The plate was incubated at 37 °C in a 5% CO_2_ environment for 4 h. After incubation, the media were removed, and the formazan crystals formed at the bottom of the wells were dissolved using DMSO for 20 min at 37 °C. The dissolved samples (200 μL) were transferred to a 96-well plate, and the absorbance was measured at 540 nm using a microplate reader (Tecan, Grödig, Austria).

### 4.4. Measurement of Melanin

B16F10 cells were seeded at a density of 8.0 × 10^4^ cells in a 60 mm dish and treated with various concentrations of the sample and 100 nM α-MSH. The cells were incubated for 72 h at 37 °C in a 5% CO_2_ incubator. After incubation, all supernatants were removed, and the cells were washed twice with cold 1× PBS. Subsequently, 200 μL of lysis buffer (1% protease inhibitor cocktail in RIPA buffer, 100:1) was added to each dish, and the mixture was incubated at 4 °C for 30 min. After incubation, the lysed cells were scraped using a cell scraper and transferred to 1.5 mL microtubes. The samples were centrifuged at 15,000 rpm for 30 min at −8 °C, and 200 μL of 1N NaOH (containing 10% DMSO) was added to the resulting pellet. The mixture was then dissolved at 80 °C for 20 min. The dissolved samples were transferred to a 96-well plate, and the absorbance was measured at 405 nm using a microplate reader.

### 4.5. Intracellular Tyrosinase Assay

B16F10 cells were seeded at a density of 8.0 × 10^4^ cells in a 60 mm dish and incubated for 24 h. The cells were then treated following the same method used for melanin content measurement. After removing the media, the cells were washed twice with cold 1× PBS, and 200 μL of lysis buffer was added per dish to lyse the cells at 4 °C for 30 min. The lysates were transferred to 1.5 mL microtubes and centrifuged at 15,000 rpm for 30 min at −8 °C. The resulting supernatants were diluted 1:10 with deionized water, and the total protein concentration was quantified using a BCA protein assay kit. Next, 20 μL of the quantified protein was mixed with 80 μL of 2 mg/mL L-DOPA (in 0.1 M sodium phosphate buffer, pH 6.8) and transferred to a 96-well plate. The absorbance at 490 nm was measured using a microplate reader at 30 min intervals for up to 1 h and 30 min at 37 °C.

### 4.6. Molecular Docking

The polyphenol oxidase (PPO) family PPO3 protein (PDB ID: 2Y9X), mushroom tyrosinase (mTYR), was obtained from the Protein Data Bank (PDB) (https://www.rcsb.org/, accessed on 10 November 2024). The protein structures were examined using PyMOL 3.0.3 software to prepare for molecular docking simulations. All compounds were sourced from PubChem (https://pubchem.ncbi.nlm.nih.gov/, accessed on 10 November 2024), and their structures were optimized using the MMFF94 force field within OpenBabel 2.4.1 software to obtain the most energetically stable conformations. Protein preparation, including the addition of hydrogen atoms, was carried out using AutoDock Tools 1.5.6, while ligands were preprocessed by hydrogenation and determination of rotatable bonds. The grid parameters for docking were defined based on the position of the co-crystallized ligand in the receptor’s structure. For the mTYR protein, the following parameters were applied: Center (X, Y, Z) = (−10.2, −30.3, −44.4), Size (X × Y × Z) = (15.0 × 15.0 × 15.0). Semi-flexible docking was performed with an exhaustiveness value of 25 using the Lamarckian genetic algorithm in AutoDock Vina 1.2.0. Finally, the docking results were visualized and analyzed using PyMOL 3.0.3 and Discovery Studio 2019 software.

### 4.7. Molecular Dynamics (MD) Simulations

Molecular dynamics (MD) simulations of PPO3 protein complexes with 8-methoxy-psoralen (8-MOP) and 5,7D-4MC were performed using GROMACS 2021. The amber 14sb force field was applied for the protein, while GAFF2 was used for the ligands. The systems were solvated using the TIP3P water model within a periodic boundary box of 1.2 nm. To maintain charge neutrality, sodium and chloride ions were added. The simulation protocol consisted of three main stages. First, energy minimization was performed using the steepest descent algorithm for 50,000 steps, stopping when the maximum force dropped below 1000 kJ/mol. This was followed by NVT equilibration for 50,000 steps at 310 K, with a 2 fs time step. Subsequently, NPT equilibration was carried out for 50,000 steps at 310 K and 1 atm pressure, also using a 2 fs time step. After energy minimization and equilibration, a 100 ns production MD simulation was performed without constraints, employing a 2 fs time step and saving coordinates every 10 ps.

### 4.8. Western Blot Analyses

B16F10 cells were seeded in 60π dishes and treated with various concentrations of the sample and 100 nM α-MSH, followed by incubation for different time periods for protein expression analysis. For the analysis of tyrosinase, TRP-1, and TRP-2 protein expression, 1.5 × 10^5^ cells/dish were seeded in 60π dishes, stabilized for 24 h, and incubated for 48 h. For the analysis of MITF and Wnt protein expression, 3.0 × 10^5^ cells/dish were seeded in 60π dishes, stabilized for 24 h, and incubated for 24 h. For the analysis of AKT protein expression, 3.0 × 10^5^ cells/dish were seeded in 60π dishes, stabilized for 24 h, treated with the sample, and incubated for 4 h. After incubation, the media were removed, and the cells were washed twice with cold 1× PBS. Subsequently, 200 μL of lysis buffer was added per well to lyse the cells at 4 °C for 30 min. The lysates were collected using a cell scraper, transferred to e-tubes, and centrifuged at 15,000 rpm for 30 min at −8 °C. The protein concentration was quantified using the Pierce™ BCA protein assay kit (Thermo Fisher Scientific). The supernatants were diluted 1:10 with deionized water, and a 1:20 dilution of the samples was transferred to a 96-well plate. BCA solution (a 50:1 mixture of reagents A and B) was added, and the mixture was incubated at 37 °C for 30 min. The absorbance was measured at 562 nm. The quantified protein samples were diluted to a concentration of 30 μg/mL and mixed with 2× Laemmli sample buffer and 2-mercaptoethanol at a 1:1 ratio. The mixture was heated at 100 °C for 5 min to prepare the loading samples. Each sample (16 μL) was loaded into the SDS–polyacrylamide gel, and electrophoresis was performed at 100 V for 30 min, followed by 200 V for 40 min to separate proteins by size. The separated proteins were transferred onto a PVDF membrane using the Trans-Blot Turbo Transfer System (Bio-Rad Laboratories, Hercules, CA, USA). The transferred membranes were washed four times with 1× TBS-T (10% Tween 20, 20× TBS buffer, deionized water at a 1:5:94 ratio) at 5 min intervals. For the detection of tyrosinase, TRP-1, TRP-2, and MITF protein expression, the membranes were blocked with 5% skim milk (in 1× TBS-T) for 1 h. For PKA, GSK-3β, and AKT protein expression, the membranes were blocked with 5% BSA (in 1× TBS-T) for 1 h, followed by washing six times with 1× TBS-T at 5 min intervals. The primary antibodies were diluted at a 1:2000 ratio in 1× TBS-T and incubated with the membranes overnight at 4 °C. The next day, the membranes were washed six times with 1× TBS-T for 5 min each, followed by incubation with the secondary antibodies diluted at a 1:1000 ratio in 1× TBS-T for 1.5 h at room temperature. The nitrocellulose membranes were then washed six times with 1× TBS-T for 5 min each. Protein bands were detected using an enhanced chemiluminescence (ECL) kit, and images were captured using a Chemidoc system (WL, Vilber Lourmat, Collégien, France). The captured bands were analyzed and quantified using ImageJ software.

### 4.9. Primary Skin Irritation Test for Human Skin

This study was conducted to evaluate the primary irritation potential of the test substance on human skin in accordance with the Personal Care Products Council (PCPC) guidelines and the Standard Operating Procedures (SOP) of Dermapro Ltd. The study protocol was reviewed and approved for ethical and scientific validity by the Institutional Review Board (IRB) of Dermapro Ltd., in accordance the principles of the Declaration of Helsinki. The participants were fully informed about the study’s purpose, procedures, and potential adverse reactions before voluntarily consenting to participate by signing a written informed consent form. The study included healthy female subjects aged 19 to 60 years without a history of dermatological disorders, and a minimum of 30 participants were recruited. Suitability for the study was confirmed through background checks and medical history assessments after selection. The test site was designated as the participant’s back, which was cleansed with 70% ethanol to ensure proper preparation. A 20 µL aliquot of the test substance was applied to the designated area using a patch, which remained attached for 24 h. Evaluations were performed 20 min and 24 h after patch removal to assess primary skin irritation responses, following PCPC guidelines for the assessment of erythema, edema, and other reactions (Table 2). The mean reaction score for each test substance was calculated using a standardized formula, and any reaction graded as +5 was considered more likely to indicate an allergic response rather than an irritation reaction; therefore, the maximum assessment grade was limited to +4 (Table 3). During all evaluation points, skin adverse reactions were assessed through interviews and observations by the examiners. If any adverse reactions occurred during the trial, the affected participants received appropriate medical treatment under the judgment and responsibility of the medical staff.

### 4.10. Statistical Analysis

All experimental results are presented as the mean ± standard deviation (SD) from at least three independent replicates. Statistical analyses were performed using WINKS SDA version 7.0.9 Professional (TexaSoft, Paris, France) and evaluated using a *t*-test to determine *p*-values. The *p*-values are indicated as * *p* < 0.05, ** *p* < 0.01, and *** *p* < 0.001.

## Figures and Tables

**Table 1:** Results of human skin primary irritation test (*n* = 32).

No	Samples	Responders	1st Assessment				2nd Assessment				Reaction Grade (R)
			+1	+2	+3	+4	+1	+2	+3	+4	
1	5,7D-4MC (50 μM)	0	0	0	0	0	0	0	0	0	0
2	5,7D-4MC (100 μM)	0	0	0	0	0	0	0	0	0	0
3	Squalene (Control)	0	0	0	0	0	0	0	0	0	0

**Table 2:** Grading system for skin primary irritation test.

Grade	Description of Clinical Observation
+1	Slight erythema
+2	Moderate erythema, possibly with barely perceptible edema at the margin, papules may be present
+3	Moderate erythema, with generalized edema
+4	Severe erythema with severe edema, with or without vesicles
+5	Severe erythema with severe edema, with or without vesicles

**Table 3:** Determination criteria for skin primary irritation.

Range of Response	Judgment
0.00 ≤ R < 0.87	None to Slight
0.87 ≤ R < 2.42	Mild
2.42 ≤ R < 3.44	Moderate
3.44 ≤ R	Severe

## Data Availability

All data generated or analyzed during this study are fully available within this published article.
